# Prognostic value of pretransplant FDG-PET in refractory/relapsed Hodgkin lymphoma treated with autologous stem cell transplantation: systematic review and meta-analysis

**DOI:** 10.1007/s00277-016-2619-9

**Published:** 2016-03-02

**Authors:** Hugo J. A. Adams, Thomas C. Kwee

**Affiliations:** Department of Radiology and Nuclear Medicine, University Medical Center Utrecht, Heidelberglaan 100, 3584 CX Utrecht, The Netherlands

**Keywords:** Autologous stem cell transplantation, FDG-PET, Hodgkin lymphoma, Meta-analysis, Systematic review

## Abstract

This study aimed to systematically review the prognostic value of pretransplant ^18^F-fluoro-2-deoxy-D-glucose positron emission tomography (FDG-PET) in refractory/relapsed Hodgkin lymphoma treated with autologous stem cell transplantation (SCT). MEDLINE was systematically searched for appropriate studies. Included studies were methodologically appraised. Results of individual studies were meta-analyzed, if possible. Eleven studies, comprising a total of 745 refractory/relapsed Hodgkin lymphoma patients who underwent FDG-PET before autologous SCT, were included. The overall methodological quality of these studies was moderate. The proportion of pretransplant FDG-PET positive patients ranged between 25 and 65.2 %. Progression-free survival ranged between 0 and 52 % in pretransplant FDG-PET positive patients, and between 55 and 85 % in pretransplant FDG-PET negative patients. Overall survival ranged between 17 and 77 % in pretransplant FDG-PET positive patients, and between 78 and 100 % in FDG-PET negative patients. Based on five studies that provided sufficient data for meta-analysis, pooled sensitivity and specificity of pretransplant FDG-PET in predicting treatment failure (i.e., either progressive, residual, or relapsed disease) were 67.2 % (95 % confidence interval [CI] 58.2–75.3 %) and 70.7 % (95 % CI 64.2–76.5 %), respectively. Based on two studies that provided sufficient data for meta-analysis, pooled sensitivity and specificity of pretransplant FDG-PET in predicting death during follow-up were 74.4 % (95 % CI 58.8–86.5 %) and 58.0 % (95 % CI 49.3–66.3 %), respectively. In conclusion, the moderate quality evidence suggests pretransplant FDG-PET to have value in predicting outcome in refractory/relapsed Hodgkin lymphoma patients treated with autologous SCT. Nevertheless, a considerable proportion of pretransplant FDG-PET positive patients remains disease free and a considerable proportion of pretransplant FDG-PET negative patients develops disease relapse after autologous SCT.

## Introduction

More than 90 % of patients with limited-stage and up to 80 % of patients with advanced-stage Hodgkin lymphoma achieve long-term disease-free survival with standard first-line therapies [1]. Unfortunately, a non-negligible fraction of Hodgkin lymphoma patients develops refractory/relapsed disease. Randomized trials have demonstrated the potential benefit of salvage chemotherapy followed by consolidation with high-dose therapy (HDT) and autologous stem cell transplantation (SCT) as second-line therapy [2, 3]. With this treatment regimen, more than 50 % of patients can be cured [2–6]. Patients who relapse after HDT and autologous SCT have dismal outcomes [7, 8]. Timely identification of non-responders to standard second-line therapy is crucial to offer them the opportunity to switch to more intensive and potentially more effective therapies such as extended salvage therapy, additional radiation therapy, allogeneic SCT, tandem SCT, or the addition of recently developed novel drugs such as brentuximab vedotin. Several parameters have proven to be predictive of poor outcome in patients with relapsed Hodgkin lymphoma, including presalvage anemia, hypoalbuminemia, lymphopenia, presence of B symptoms, extranodal involvement at relapse, Karnofsky score <90 %, and a time interval between first-line therapy and relapse detection of less than 1 year [4, 9–12]. Another method that might provide prognostic information is ^18^F-fluoro-2-deoxy-D-glucose positron emission tomography (FDG-PET). FDG-PET is widely used for staging Hodgkin lymphoma [13, 14], but may also be performed during treatment to identify non-responders. In newly diagnosed Hodgkin lymphoma, FDG-PET after 1–4 cycles of first-line therapy has shown to have prognostic value, although results are heterogeneous among different studies [15]. Over the past few years, several studies have also evaluated the prognostic value of pretransplant FDG-PET in refractory/relapsed Hodgkin lymphoma patients undergoing autologous SCT. However, there may be variability among individual studies with regard to internal validity (i.e., risk of bias) and external validity (i.e., generalizability of study results). A systematic review and meta-analysis are required to better comprehend the value of FDG-PET in this setting. The purpose of this study was therefore to systematically review and meta-analyze published data on the prognostic value of pretransplant FDG-PET in patients with refractory/relapsed Hodgkin lymphoma treated with salvage therapy, HDT, and autologous SCT.

## Materials and methods

### Search strategy

Medline was searched using the PubMed interface for original studies on the prognostic value of pretransplant FDG-PET in refractory/relapsed Hodgkin lymphoma, from start date to 12 July 2015. The search strategy is displayed in Table 1. References of the included studies were scrutinized for suitable references that were not retrieved by the initial PubMed search.

**Table 1:** Medline search performed using the PubMed interface on 12 July 2015

No.	Search string	No. of hits in Medline
1.	Fluorodeoxyglucose or 2-fluoro-2-deoxy-D-glucose or FDG or 18F-FDG or positron emission tomography or PET	89,372
2.	Hodgkin or Hodgkin’s or Hodgkins	81,948
3.	No. 1 and no. 2	1768

### Study selection

Original studies reporting on the value of pretransplant FDG-PET in predicting patient outcome (either number of progressive, residual, or relapsed diseases, number of deaths, progression-free survival, or overall survival) in refractory/relapsed Hodgkin lymphoma treated with salvage therapy, HDT, and autologous SCT were eligible for inclusion. Studies were excluded if not written in English, Spanish, French, German, Italian, or Dutch. Articles without original patient data, such as reviews, editorials, letters, and conferences abstracts, were excluded. Articles with less than 10 patients and articles from which the same patient data were used in a more recent article were also excluded. Articles in which patients with Hodgkin lymphoma could not be separated from patients with other lymphoma subtypes, articles in which patients receiving allogeneic SCT could not be separated from patients receiving autologous SCT, and articles in which patients undergoing FDG-PET could not be separated from patients who were examined with other imaging techniques (e.g., gallium scans) were excluded. Articles in which therapy was modified on the basis of the pretransplant FDG-PET result (except for patients who were allocated to another salvage regimen in case of evident non-response or disease progression during salvage treatment, which is considered reflective of clinical practice) were not included into the main analysis, but addressed separately. Titles and abstracts of all studies that were obtained by the PubMed search were screened using these inclusion and exclusion criteria. Articles that were certainly ineligible were excluded at this stage. The remaining articles were then retrieved in full-text format to make a final decision as to whether they met the criteria to be included in this systematic review and meta-analysis.

### Methodological quality assessment

The Quality in Prognosis Studies (QUIPS) criteria were used to assess the methodological quality of included studies [16]. The QUIPS criteria include six different domains: risk of bias in study participation (“do the study data available [i.e., patients not lost to follow-up] adequately represent the study sample?”), prognostic factor measurement (“is the prognostic factor measured in a similar way for all participants?”), outcome measurement (“is the outcome of interest measured in a similar way for all participants?”), study confounding (“have important potential confounding factors appropriately been accounted for?”), and statistical analysis and reporting (“is the statistical analysis appropriate, and are all primary outcomes reported”) [16]. Risk of bias was scored as low, moderate, or high for each of these six domains [16].

### Meta-analysis

Studies were eligible for meta-analysis if they provided sufficient data to construct 2 × 2 contingency tables to calculate the sensitivity and specificity of pretransplant FDG-PET in predicting treatment failure (i.e., either progressive, residual, or relapsed disease) and/or death during follow-up. This meta-analysis did not include studies that applied pretransplant FDG-PET-adapted therapies. To explore whether using different thresholds in studies included may have affected sensitivity or specificity, Spearman *ρ* (comparison of the logit of the sensitivity and logit of 1-specificity) was calculated. Spearman *ρ* > 0.6 was considered to demonstrate the presence of a threshold effect [17]. If a threshold effect was absent, summary estimates of sensitivity and specificity were calculated, using the DerSimonian and Laird method [18]. The Higgins and Thompson test was used to assess heterogeneity in diagnostic odds ratios (DORs) across individual studies [19]. Heterogeneity was defined as *I*^2^ exceeding 50 %. The DOR is an overall measure of accuracy of a diagnostic test that is not dependent of the threshold value or disease prevalence [20].

## Results

### Literature search

The Medline search revealed 1768 articles (Table 1). After screening titles and abstracts, 30 remained. Of these, 5 were excluded because these studies did not allow separate data extraction of patients with Hodgkin lymphoma from those with other non-Hodgkin lymphoma subtypes, 3 were excluded because these studies included less than 10 patients with Hodgkin lymphoma, 3 were excluded because these studies reported insufficient data about the prognostic consequences of the pretransplant FDG-PET status, 2 were excluded because these studies mixed pretransplant FDG-PET and gallium scan findings, 1 was excluded because patients undergoing autologous SCT could not be separated from patients undergoing allogeneic SCT, 1 was excluded because only patients with complete remission at pretransplant FDG-PET were included, and 1 was excluded because it included a group of patients that was treated without SCT and who could not be separated from patients undergoing autologous SCT. Finally, 14 articles, of which 11 did not change the therapy on the basis of the pretransplant FDG-PET results, and 3 applied pretransplant FDG-PET-adapted therapy, were included. The characteristics of these studies are displayed in Tables 2 and 3.

**Table 2:** Characteristics of included studies and patients

Study (year)	Country	Data acquisition	No. of patients	Age in years (range)	Sex (M/F)	Stage at relapse (No.)	Salvage regimens	HDT	Primary regimens
Gentzler et al. [21] (2014)	USA	Retrospective	51^a^	35^b^ (18–67)	28/23	II: 17 III: 16 IV: 17	ESHAP: 23 ICE: 19 MOPP ± ESHAP: 4 Other: 5	CCE	MOPP ± ABVD: 7 ABVD: 41 Other: 3
Nieto et al. [22] (2013)	USA	Prospective	180^c^	32^b^ (17–65)	106/74	NR	First-line therapy salvage: ICE: 101 ESHAP: 54 IGEV: 25 Second-line therapy: GVD: 26 IGEV: 24 ICE: 20 Other: 8 Third-line therapy: GVD: 18 IGEV: 17 ICE: 8 Other: 10	Gem-Bu-Mel: 84 BEAM: 57 Bu-Mel: 39	ABVD: 176 Stanford V: 3 Other: 1
Cocorocchio et al. [23] (2013)	Italy	Retrospective	97^d^	Median 34 (18–66)	49/48	I: 2 II: 52 IIIA: 13 IVA: 30	ESHAP: 73 IGEV: 16 DHAP: 8	BEAM: 84 idarubicin + melphalan: 13	NR
Akhtar et al. [24] (2013)	Saudi Arabia	Prospective	141	25.5^b^ (15–60)	78/63	I-II: 48 III-IV: 93	ESHAP: 133 Other: 8	BEAM	ABVD: 129 Other: 12
Sucak et al. [25] (2011)	Turkey	Retrospective	43	30^b^ (16–61)	34/9	II: 13 III: 11 IV: 19	GVD: 30 ICE: 7 Other: 6	BEAM	NR
Smeltzer et al. [26] (2011)	USA	Retrospective	46	36^b^ (18–70)	27/19	I: 4 II: 25 III: 6 IV: 9	ESHAP: 19 ICE: 15 GVD: 5 Other: 6	BEAM	ABVD: 36 Stanford V: 5 Other: 5
Mocikova et al. [27] (2011)	Czech Republic	Retrospective	76	34^b^ (20–61)	40/36	I-II: 26 III-IV: 50	DHAP/ESHAP: 41 ICE: 15 CEVD: 10 ABVD: 6 Other: 4	BEAM: 72 MM + BEAM (tandem SCT): 4	NR
Arai et al. [28] (2010)	USA	Prospective	92^e^	33^b^ (18–64)	48/44	NR	DHAP: 40 ICE: 35 Other 17	GV + reduced BCNU	ABVD 60 Stanford V: 32
Castagna et al. [29] (2009)	Italy	Retrospective	24	35^b^ (15–51)	16/8	I-II: 12 III-IV: 12	IGEV	NR	NR
Jabbour et al. [30] 2007	USA	Retrospective	211^f^	30^b^ (11–77)	124/87	I: 6 II: 91 III: 60 IV: 54	ESHAP/ASHAP: 171 Other: 40	BEAM: 145 CBV or BuCy: 66	ABVD: 164 Other: 47
Schot et al. [31] (2007)	Netherlands	Prospective	101^g^	53 (19–68)	59/42	I/II: 33 III/IV: 68	DHAP-VIM-DHAP	BEAM	NR
Pretransplant FDG-PET-adapted therapeutic trials									
Moskowitz et al. [32] (2015)	USA	Prospective	45	31^b^ (13–65)	25/20	I: 1 II: 14 III: 11 IV: 12	Brentuximab vedotin (+ICE^h^)	BEAM: 22 CBV: 14 TLI + CE: 7 Other: 1	ABVD: 41 BEACOPP: 3 R-CHOP/CEPP: 1
Devillier et al. (2012) [33]	Multinational	Retrospective	111	33^b^ (17–71)	65/46	I/II: 50 III/IV: 58 Unknown: 3	DHAP IVA ICE	BEAM: BEAM+ NCBV (tandem SCT):	NR
Moskowitz et al. (2012) [34]	USA	Prospective	97	35^b^ (19–72)	41/56	NR	ICE/augmented ICE (+GVD^h^)	CBV or CE + RT	ABVD: 79 Other: 18

*Abbreviations*: *ABVD* adriamycin, bleomycin, vinblastine, and dacarbazine, *ASHAP* doxorubicin, methylprednisolone, ara-c, cisplatin, *BEAM* carmustine, etoposide, ara-c, melphalan, *BuCy* busulfan, cyclophosphamide, *CBV* cyclophosphamide, carmustine, etopside, *CCE* cyclophosphamide, carboplatin, etoposide, *CE* cyclophosphamide, etoposide, *DHAP* dexamethasone, ara-c, cisplatin, *ESHAP* etoposide, methylprednisolone, ara-c, cisplatin, *Gem-Bu-Mel* gemcitabine, busulfan, melphalan, *GVD* gemcitabine, vinorelbine, doxil, *ICE* ifosfamide, carboplatin, etoposide, *IVA* ifosfamide, etoposide, doxorubicin, *IGEV* ifosfamide-gemcitabine-vinorelbine, *MM* melphalan and mitoxantrone, *MOPP* mechlorethamine, vincristine, procarbazine, and prednisone, *NR* not reported, *R*-*CHOP*/*CEPP* rituximab, cyclophosphamide, vincristine, prednisone, etoposide, and procarbazine, *VIM* etoposide, ifosfamide, methotrexate, *TLI* total lymphoid irradiation

^a^32 of 51 included patients underwent pretransplant FDG-PET

^b^Median

^c^175 of 180 included patients underwent pretransplant FDG-PET

^d^40 of 97 included patients underwent pretransplant FDG-PET

^e^77 of 92 included patients underwent pretransplant FDG-PET

^f^68 of 211 included patients underwent pretransplant FDG-PET

^g^23 of 101 included patients underwent pretransplant FDG-PET and were diagnosed with Hodgkin lymphoma

^h^Additional therapy in case of an FDG-PET positive result

**Table 3:** FDG-PET imaging and interpretation methods, and criteria for treatment failure that were used in the included studies

Study (year)	Imaging system(s)	Interpreters	Histological verification of relapse/refractory disease before salvage therapy	Histological verification of relapse/refractory disease after autologous SCT
Gentzler et al. [21] (2014)	NR	Nuclear medicine radiologist	Yes (all cases)	NR
Nieto et al. [22] (2013)	NR	NR	NR	NR
Cocorocchio et al. [23] (2013)	NR	NR	NR	NR
Akhtar et al. [24] (2013)	Stand-alone or hybrid FDG-PET/CT	Two nuclear medicine physicians	Yes: 91 No: 50	NR
Sucak et al. [25] (2011)	Hybrid FDG-PET/CT	Two nuclear medicine physicians	NR	NR
Smeltzer et al. [26] (2011)	Hybrid FDG-PET/CT	Two nuclear medicine physicians	NR	NR
Mocikova et al. [27] (2011)	Stand-alone FDG-PET or hybrid FDG-PET/CT	Local nuclear medicine report	Yes (all cases)	NR
Arai et al. [28] (2010)	Hybrid FDG-PET/CT	NR	Yes (all cases)	NR
Castagna et al. [29] (2009)	Stand-alone FDG-PET	Experienced nuclear medicine physician	NR	NR
Jabbour et al. [30] (2007)	Stand-alone FDG-PET or hybrid FDG-PET/CT	NR	Yes (all cases)	NR
Schot et al. [31] (2007)	Stand-alone FDG-PET	Two independent reviewers	Yes (all cases)	NR
Pretransplant FDG-PET-adapted therapeutic trials				
Moskowitz et al. [32] (2015)	Hybrid PET/CT	Nuclear medicine physician	Yes (all cases)	NR
Devillier et al. (2012) [33]	Hybrid PET/CT	Nuclear medicine physician	Yes	NR
Moskowitz et al. (2012) [34]	NR	Nuclear medicine physician	Yes (all cases)	NR

*NR* not reported

### Methodological quality

The methodological quality assessment using the QUIPS criteria is displayed in Table 4. Overall, the methodological quality was moderate. There was moderate risk of bias for the domain of study inclusion in six studies, because five studies [22, 23, 25, 26, 29] did not report whether refractory/relapsed disease was histologically confirmed before initiation of salvage therapy and one study [24] reported that refractory/relapsed disease was not histologically verified in 50/141 patients. There was moderate risk of bias for the domain of prognostic factor measurement in six studies, because four studies did not report the whether a stand-alone PET system or integrated PET/CT system was used [21–23, 34], and two studies [24, 29] included patients who underwent stand-alone FDG-PET. In addition, there was high risk of bias for the domain of prognostic factor measurement in three studies [27, 30, 31], because these studies used stand-alone FDG-PET systems and did not use standardized international FDG-PET interpretation criteria. There was moderate risk of bias in all 14 included studies, because none of these studies reported that refractory/relapsed disease after autologous SCT was histologically verified. Finally, there was moderate risk of bias for study confounding in 12 of 14 studies [21–28, 30, 32–34], because these studies included patients treated with heterogeneous treatment regimens.

**Table 4:** Quality assessment of included studies (risk of bias in six different domains according to the QUIPS tool [16])

Study (year)	Study participation	Study attrition	Prognostic factor measurement	Outcome measure	Study confounding	Statistical analysis
Gentzler et al. [21] (2014)	Low	Low	Moderate	Moderate	Moderate	Low
Nieto et al. [22] (2013)	Moderate	Low	Moderate	Moderate	Moderate	Low
Cocorocchio et al. [23] (2013)	Moderate	Low	Moderate	Moderate	Moderate	Low
Akhtar et al. [24] (2013)	Moderate	Low	Moderate	Moderate)	Moderate	Low
Sucak et al. [25] (2011)	Moderate	Low	Low	Moderate	Moderate	Low
Smeltzer et al. [26] (2011)	Moderate	Low	Low	Moderate	Moderate	Low
Mocikova et al. [27] (2011)	Low	Low	High	Moderate	Moderate	Low
Arai et al. [28] (2010)	Low	Low	Low	Moderate	Moderate	Low
Castagna et al. [29] (2009)	Moderate	Low	Moderate	Moderate	Low	Low
Jabbour et al. [30] (2007)	Low	Low	High	Moderate	Moderate	Low
Schot et al. [31] (2007)	Low	Low	High	Moderate	Low	Low
Pretransplant FDG-PET-adapted therapeutic trials						
Moskowitz et al. [32] (2015)	Low	Low	Low	Moderate	Moderate	Low
Devillier et al. (2012) [33]	Low	Low	Low	Moderate	Moderate	Low
Moskowitz et al. (2012) [34]	Low	Low	Moderate	Moderate	Moderate	Low

### Prognostic value pretransplant FDG-PET

Results of the 11 studies on the prognostic value of pretransplant FDG-PET are shown in Table 5. The proportion of pretransplant FDG-PET positive patients ranged between 25 and 65.2 %. Progression-free survival ranged between 0 and 52 % in pretransplant FDG-PET positive patients, and between 55 and 85 % in pretransplant FDG-PET negative patients. Overall survival ranged between 17 and 77 % in pretransplant FDG-PET positive patients, and between 78 and 100 % in FDG-PET negative patients.

**Table 5:** Results of included studies

Study (year)	Follow-up time in months (range)	Interpretation criteria	No. of pretransplant FDG-PET positive and negative patients (%)	Proportion of patients experiencing treatment failure in interim FDG-PET positive group	Proportion of patients experiencing treatment failure in interim FDG-PET negative group	Proportion of patients experiencing in interim FDG-PET positive group	Proportion of patients experiencing death in interim FDG-PET negative group	PFS	Hazard ratio PFS FDG-PET negative vs. positive	OS	Hazard ratio OS FDG-PET negative vs. positive
Gentzler et al. [21] (2014)	38^a^	Deauville 0–2 vs. 3–5 [13]	Pos: 19 (59 %) Neg: 13 (41 %)	NR	NR	NR	NR	5-year PFS: Pos: 52 % Neg: 85 %	NR	5-year OS: Pos: 48 % Neg: 100 %	NR
Nieto et al. [22] (2013)	36^a^ (3–72)	IHP [35]	Pos: 69 (39.4 %) Neg: 106 (60.6 %)	NR	NR	NR	NR	EFS: Pos: 32 % Neg: 55 %	NR	OS: Pos: 49 % Neg: 78 %	NR
Cocorocchio et al. [23] (2013)	45^a^ (1–164)	Gallamini criteria [36]	Pos: 14 (35 %) Neg: 26 (65 %)	8/14 (57.1 %)	5/26 (19.2 %)	7/14 (50 %)	3/26 (11.5 %)	Estimated 5-year PFS: Pos: 43 % Neg: 79 %	NR	Estimated 5-year OS: Pos: 43 % Neg: 92 %	NR
Akhtar et al. [24] (2013)	33^a^ (12–81)	IHP [35]	Pos: 76 (53.9 %) Neg: 65 (46.1 %)	37/76 (48.7 %)	10/65 (15.4 %)	25/76 (32.9 %)	8/65 (12.3 %)	NR	4.1	NR	3.9
Sucak et al. [25] (2011)	21.5^a^ (6–64.5)	IHP [35]	Pos: 26 (60.5 %) Neg: 17 (39.5 %)	NR	NR	NR	NR	PFS: Pos: 37.6 % Neg: 70.6 %	3.91	OS Pos: 74.5 % Neg: 94.1 %	NR
Smeltzer et al. [26] (2011)	37.8^b^	IHP [35]	NR	NR	NR	NR	NR	PFS: Pos: 41 % Neg: 82 %	3.2 (95 % CI 1.1–9.0)	OS: Pos: 64 % Neg: 91 %	3.6 (95 % CI 0.75–17.5)
Mocikova et al. [27] (2011)	33.6^a^ (3.2–105.8).	No international standardized criteria	Pos: 20 (26.3 %) Neg: 56 (73.7 %)	11/20 (55.0 %)	14/56 (25.0 %)	NR	NR	Estimated 2-year PFS: Pos: 36.1 % Neg: 72.7 %	2.68	Estimated 2-year OS: Pos: 61.4 % Neg: 90.3 %	3.47
Arai et al. [28] (2010)	29^a^ (8–86)	Gallamini criteria [36]	Pos: 29 (37.7 %) Neg: 48 (62.3 %)	NR	NR	NR	NR	2-year PFS: Pos: 45 % Neg: 79 %	3.3	2-year OS: Pos: 77 % Neg: 91 %	NR
Castagna et al. [29] (2009)	24^a^	IHP [35]	Pos: 6 (25 %) Neg: 18 (75 %)	NR	NR	NR	NR	2-year PFS: Pos: 0 % Neg: 78 %	10.1	2-year OS: Pos: 17 % Neg: 87 %	13.3
Jabbour et al. [30] (2007)	33.6^a^	No international standardized criteria	Pos: 25 (36.8 %) Neg: 43 (63.2 %)	18/25: (72.0 %)	10/43: (23.3 %)	NR	NR	NR	NR	NR	NR
Schot et al. [31] (2007)	22^a^ (6–61).	No international standardized criteria	Pos: 15 (65.2 %) Neg: 8 (34.8 %)	10/15 (66.7 %)	2/8 (25.0 %)	NR	NR	Estimated 2-year PFS: Pos: 38 % Neg: 73 %	NR	NR	NR
Pretransplant FDG-PET-adapted therapeutic trials											
Moskowitz et al. [32] (2015)	20.1^a^	Deauville 0–2 vs. 3–5 [13]		NR	NR	NR	NR	2-year PFS: Pos + Pos: 46 % Pos + Neg: 91 % Neg + Neg: 92 %	5.7	NR	NR
Devillier et al. (2012) [33] (single SCT cohort)	36^a^	IHP [35]	Pos: 12 Neg: 35	NR	NR	NR	NR	5-year PFS: Pos: 0 % Neg: 75 %	NR	5-year OS: Pos: 47 % Neg: 84 %	NR
Devillier et al. (2012) [33] (tandem SCT cohort)	36^a^	IHP [35]	Pos: 14 Neg: 50	NR	NR	NR	NR	5-year PFS: Pos: 43 % Neg: 87 %	NR	5-year OS: Pos: 56 % Neg: 93 %	NR
Moskowitz et al. (2012) [34]	51^a^ (16–86)	>Mediastinal/abdominal aortic bloodpool	Pos + Pos: 21 Neg + Pos: 17 Neg: 59	NR	NR	NR	NR	Estimated 4-year PFS: Pos + Pos: ±30 % Pos + Neg: ±80 % Neg: ±80 %	4.6	NR	NR

*Abbreviations*: *EFS* event-free survival, *IHP* international harmonization project, *Neg* FDG-PET negative after standard therapy, *NR* not reported, *OS* overall survival, *PFS* progression-free survival, *Pos* FDG-PET positive, *Pos + Neg* positive after standard therapy, negative after FDG-PET-directed therapy, *Pos + Pos* positive after standard and FDG-PET-directed therapy

^a^Median

^b^Mean

Based on five studies that provided sufficient data for meta-analysis, pooled sensitivity and specificity of pretransplant FDG-PET in predicting treatment failure (i.e., either progressive, residual, or relapsed disease) were 67.2 % (95 % confidence interval [CI] 58.2–75.3 %) and 70.7 % (95 % CI 64.2–76.5 %), respectively. Note that there was no threshold effect (Spearman *ρ* = 0.6, *P* = 0.285) and no heterogeneity in DORs among these five studies (*I*^2^ = 0.0 %).)

Based on two studies that provided sufficient data for meta-analysis, pooled sensitivity and specificity of pretransplant FDG-PET in predicting death were 74.4 % (95 % CI 58.8–86.5 %) and 58.0 % (95 % CI 49.3–66.3 %), respectively. Note that the presence of a threshold effect and heterogeneity in DORs could not statistically be assessed with only two studies.

### Pretransplant FDG-PET-adapted therapeutic studies

Three studies were included that applied FDG-PET-adapted therapy. Two studies [32, 34] directed the therapy in a systematic fashion (applied extended lines of salvage therapy in FDG-PET positive patients) and showed that patients who acquired FDG-PET negative status after extended lines of salvage therapy (91 and ±80 %, respectively) had a similar PFS as those who acquired FDG-PET negative status after standard therapy (92 % and ±80, [32, 34]. On the other hand, patients who did not acquire FDG-PET negative status had a worse prognosis (46 and ± 30 %, [32, 34]. One retrospective study [33] applied tandem autologous SCT in high-risk patients (where risk was determined on the basis of the pretransplant FDG-PET result and other factors [33]). Patients with pretransplant FDG-PET positive status treated with tandem autologous SCT had a better 5-year PFS (43 %) than those treated with single autologous SCT (0 %), whereas this benefit in terms of 5-year PFS was less clear in those with a negative pretransplant FDG-PET (87 vs. 75 %, respectively [33]).

## Discussion

The present systematic review and meta-analysis show that the patients with a pretransplant FDG-PET scan that is positive for residual disease generally have a worse outcome (both in terms of progression-free survival and overall survival) than those with a negative pretransplant FDG-PET scan. The predictive value of pretransplant FDG-PET appears to be moderate, however, with pooled sensitivity and specificity pairs of 67.2 and 70.7 % for the prediction of treatment failure, and 74.4 and 58.0 % for predicting death during follow-up. Since both false-positives and false-negatives are not uncommon, it is questionable whether incorporating pretransplant FDG-PET results in treatment planning in routine clinical practice is justified at this moment.

Several prediction rules have been developed to predict outcome in patients with relapsed/refractory Hodgkin lymphoma undergoing autologous SCT: the Memorial Sloan-Kettering Cancer Center (MSKCC) prognostic model [10], the Grupo Espanol de Linfomas/Trasplante Autologo de Medula Osea (GEL/TAMO) score [4], the Simplified Validated Prognostic Model of the Center for International Blood and Marrow Transplant Research (CIBMTR) [12], and the Adapted Prognostic Score which was developed using the cohort of the HDR2 trial [37]. The MSKCC found the three factors duration of remission <1 year, presence of extranodal disease before salvage therapy, and presence of B symptoms before salvage therapy, to be significantly predictive of outcome after autologous SCT [10]. With a median follow-up of 43 months, progression-free survival was 83 % for patients with zero or one risk factor, 27 % for patients with two risk factors, and 10 % for patients with all three risk factors [10]. The GEL/TAMO group identified the following prognostic factors: presence of extranodal disease, chemotherapy refractory disease, and duration of complete remission <12 months prior to relapse [4]. Patients with 0–1, 2, and 3 risk factors had long-term progression-free survival rates of 71, 51, and 18 %, respectively [4]. The CIBMTR prognostic model [12] found three significant independent predictive factors in the multivariate analysis and developed the following risk score: Karnofsky performance score <90 and chemotherapy resistance at autologous SCT were assigned one point, and three or more chemotherapy regimens before autologous SCT and extranodal disease present at autologous SCT were assigned two points. Based on the sum score for the four risk factors, three groups were identified: low (0 points), intermediate (1–3 points), or high (4–6 points). The 4 years progression-free survival for the low, intermediate, and high risk groups were 71, 60, and 42 %, respectively [12]. Finally, the Adapted Prognostic Score of the HDR2 trial [37] identified the risk factors presence of stage IV at relapse, anemia, and early or multiple relapse. Patients with 0, 1, 2, and 3 of these risk factors had a 3-year progression-free survival of approximately 80, 70, 50, and 15 %, respectively. Future studies are required to compare pretransplant FDG-PET to these clinical risk assessment models and to assess whether the former has any additional value to the latter.

Three prospective studies in which the treatment strategy was based on the pretransplant FDG-PET result have been published. A study by Moskowitz et al. [34] published in 2012 included patients with histologically proven refractory/relapsed Hodgkin lymphoma who were treated with salvage ifosfamide, carboplatin, etoposide (ICE) chemotherapy. After salvage chemotherapy, patients underwent a restaging FDG-PET. Positive scans were defined by site of disease as follows: supradiaphragmatic Hodgkin lymphoma, FDG uptake greater than mediastinal blood pool; and infradiaphragmatic Hodgkin lymphoma, FDG uptake greater than abdominal aortic blood pool. If the FDG-PET scan was negative, patients proceeded to radiotherapy, HDT, and autologous SCT, whereas patients with positive FDG-PET scans were additionally treated with 2 cycles of gemcitabine, vinorelbine and liposomal doxorubicin (GVD), followed by a second restaging FDG-PET scan, radiotherapy, HDT, and autologous SCT. Fifty-eight (60 %) of patients received an FDG-PET based complete response after ICE chemotherapy. Of the remaining 36 patients, 33 were treated with additional GVD, of whom 17 acquired a post GVD negative FDG-PET status. The event-free survivals (EFSs) of patients who achieved a negative FDG-PET after ICE or GVD were almost similar (estimated 4-year EFS approximately 80 %), whereas those who did not acquire a FDG-PET negative status had a worse prognosis (estimated 4-year EFS 28.6 %). Moskowitz et al. [34] concluded that their study provides evidence that the goal of salvage therapy in patients with Hodgkin lymphoma should be a negative FDG-PET scan before HDT and autologous SCT. Unfortunately, their study did not report the effect of their treatment strategy and/or FDG-PET status on the overall survival. Consequently, it still remains unclear whether this treatment strategy will result in a long-term benefit in overall survival. Patients with post HDT and autologous SCT residual disease may have been cured with third-line therapies, thereby relatively decreasing the survival benefit of Moskowitz et al.’s [34] treatment strategy. On top of that, a third-line therapy only exposed the patients in whom second-line therapy failed to additional therapies, and not the entire group of post-salvage FDG-PET positive patients. Of note, 5 of 38 post ICE positive patients became transplant ineligible after the GVD courses, and this number might have been lower when HDT and autologous SCT were applied directly after the ICE/augmented ICE courses. Finally, Moskowitz et al. [34] did not report whether residual disease after autologous SCT was histologically verified. Another study by Moskowitz et al. [32] published in 2015 included 45 patients who were treated with 2 cycles of brentuximab vedotin as first-line salvage treatment, followed by an FDG-PET scan, which was positive in 33 and negative in 12 patients. Positive patients were treated with second-line salvage therapy by ICE whereas negative patients were directly treated with HDT and autologous SCT. Thirty-two of 33 patients had FDG-PET scans after salvage ICE, of whom 22 were negative and 10 remained positive, followed by HDT and autologous SCT in all 32 cases. The median follow-up was 20 months. At 2 years, EFS of patients who were FDG-PET negative after brentuximab vedotin therapy was 92 %, and that of those who were FDG-PET negative after brentuximab vedotin and additional ICE was 91 %. Patients who were still FDG-PET positive after brentuximab and vedodotin and ICE had an EFS of 46 %. Unfortunately, this study did not report the influence of the FDG-PET status on the overall survival either. Finally, Devillier et al. [33] performed a retrospective analysis of 111 patients with relapsed/refractory Hodgkin lymphoma. Pretransplant FDG-PET status was considered in order to select subsequent therapy by means of either HDT with autologous SCT or tandem HDT with autologous SCT. The choice of single or tandem transplantation was made on the basis of both risk factors (interval from end of first-line therapy to relapse <12 months; Ann-Arbor stage III or IV at relapse; and relapse in a previously irradiated field) at relapse and PET response after salvage therapy. After salvage therapy, 85 patients acquired pretransplant FDG-PET negative status, of whom 50 underwent tandem autologous SCT, and 26 acquired pretransplant FDG-PET positive status, of whom 12 underwent tandem autologous SCT. Five-year progression-free survival and overall survival were 75 and 84 % in pretransplant FDG-PET negative, and 0 and 47 % in pretransplant positive patients treated with a single HDT and autologous SCT, respectively. On the other hand, 5-year progression-free survival and overall survival were 87 and 93 % in pretransplant negative, and 43 and 56 % in pretransplant FDG-PET positive patients treated with tandem HDT and autologous SCT. In other words, particularly pretransplant FDG-PET positive patients appeared to benefit from tandem HDT and autologous SCT. However, it should be realized that this study included a low number of pretransplant FDG-PET positive patients and that other risk factors were considered in the determination of the treatment strategy. Thus, although the first results of FDG-PET adapted trials are encouraging, the survival benefit of this treatment strategy has not convincingly been proven.

The present systematic review and meta-analysis had several limitations. First, methodological quality of studies included was moderate. Particularly the inter- and intrastudy variability in salvage regimens and number of therapy cycles might have influenced the predictive value of pretransplant FDG-PET. During first-line therapy, the predictive value of interim FDG-PET has already been shown to be influenced by pretreatment risk factors and the treatment regimen that is used [15]. It is not unlikely that these results can be extrapolated to the second-line therapy setting. Second, although studies changing treatment strategy on the basis of the pretransplant FDG-PET result were separated from those who did not apply pretransplant FDG-PET-adapted therapy, it cannot be excluded that some of the included studies might have applied FDG-PET based therapies without clearly describing this in their methodology. Particularly in retrospective studies, the FDG-PET result might have driven clinicians to alter treatment planning (e.g., additional cycles of salvage therapy) and order additional FDG-PET scans before the actual autologous SCT. Third, only five studies reported sufficient data for a meta-analysis on the value of pretransplant FDG-PET in predicting treatment failure, and only two studies reported sufficient data for a meta-analysis on the value of pretransplant FDG-PET in predicting overall survival. Fourth, data on interobserver agreement in FDG-PET interpretation were not reported by individual studies. Interobserver variability of FDG-PET interpretation during therapy (kappa values of 0.66–0.84 have been reported in such settings [38, 39]) may have affected the results of both individual studies and this meta-analysis.

In conclusion, the moderate quality evidence suggests pretransplant FDG-PET to have value in predicting outcome in refractory/relapsed Hodgkin lymphoma patients treated with autologous SCT. Nevertheless, a considerable proportion of pretransplant FDG-PET positive patients remains disease free and a considerable proportion of pretransplant FDG-PET negative patients develops disease relapse after autologous SCT.
